# Chocolate and Broma

**Published:** 1850-01

**Authors:** 


*Chocolate and Broma.*—Broma, an admirable preparation, alike agreeable to the well or sick, has acquired a reputation which we think it certainly deserves. A few years since, a great manufacturer of broma, Mr. Baker, of Dorchester, Mass., sought the opinions of many medical gentlemen of distinction, for the purpose of having an unobjectionable food for invalids, and he was assured that he had fully succeeded. Hospitals, infirmaries, and households generally, should always be provided with it. When gruel, arrowroot, groats, barley, starch, rice, farina, and many other things ordinarily resorted to for patients are of no utility, the broma is sometimes relished. It is believed that those who use it as a daily beverage will have manifest dietetic advantages over the consumers of tea and coffee. We see it stated that during the last summer those individuals who were habitually using chocolate or broma, neither had attacks of cholera or dysenteric affections, while others in the same families, taking their daily potations of tea, coffee, or simple cold water, where the sufferers, if any. We cannot vouch for the truth of this, but it has recalled to mind the statement that the oil dealers in London have been free from cholera or the choleroid symptoms. And it has been further observed here in Boston, that persons who were taking cod-liver oil for chronic difficulties, during the prevalence of the late epidemic, were not affected by it. Vegetable oil in the first instance, and animal oil in the last, taken internally, would appear, by these statements, to have secured those who took them from the shafts of the pestilence. It is certainly a point well worth while to determine, whether the chocolate drinkers have been secure in other infected cities.—*Boston Med. and Surgical Journal.*

